# Production of eco friendly DME fuel over sonochemically synthesized UiO66 catalyst

**DOI:** 10.1038/s41598-024-52155-8

**Published:** 2024-01-19

**Authors:** Mahdi Sharifi, Rouein Halladj, Sima Askari

**Affiliations:** 1 Department of Chemical Engineering, Amirkabir University of Technology (Tehran Polytechnic), Tehran, Iran; 2 Department of Chemical Engineering, Science and Research Branch, Islamic Azad University, Tehran, Iran

**Keywords:** Chemistry, Materials science

## Abstract

The ultrasound-assisted preparation of UiO-66 was carried out at T = 80–220 °C, and the catalytic performances were evaluated in methanol conversion. Also, physicochemical properties were assessed by XRD, SEM, PSD, FTIR, N_2_ adsorption–desorption, TG-DTG, and NH_3_-TPD analysis. The characterization proved that increasing the synthesis temperature positively affected the crystallinity, specific surface area, thermal stability, and acidity of the catalysts. Besides, the catalysts' performance was investigated in the methanol conversion reaction (T = 350–450 °C, P = 1 atm, and WHSV = 5 h^−1^), leading to the DME (Dimethyl Ether) production. Rising reaction temperature increased the methanol conversion and DME yield. The synthesized sample at 220 °C had the best properties and performance with conversion and yield of about 38% and 51%, respectively. The stability test for the UiO-66-220 (University of Oslo 66) catalyst was performed at 450 °C for 12 h, and the activity remained stable for about 5 h. Furthermore, the used catalyst was characterized via XRD and TG analysis.

## Introduction

Metal–organic frameworks (MOFs) or porous coordinate polymers (PCPs) are interesting three-dimensional crystalline porous materials. Due to their various and unique properties, such as large surface areas, uniform but tunable pore size, and tailored chemical environment, they have attracted extensive attention recently^1–5^. Combining these essential characteristics makes MOFs promising materials for wide potential applications in drug delivery, gas storage, separation, and catalysis^3–10^.

Despite the unique properties of these materials, the catalytic activity, thermal, and chemical stabilities of them have been challenged^11–14^. Lewis et al.^1^ investigated the hydrothermal and chemical stability of widely used MOFs in a systematic study. The results showed that UiO-66 (Zircona-MOFs family) was more stable than other compounds, owing to strong Zr-O bonds. Besides, various studies have also demonstrated that UiO-66 has thermal stability in the range of 500–550 °C^15–17^. UiO-66 has two strong and weak acidic sites with different compositions^17–19^. Nevertheless, it is not commonly known as a strong acid catalyst. Therefore, special attention has been paid to increasing its acidity and catalytic activity^20–22^. Various studies have revealed that creating defects in the structure is an effective method to improve the catalytic properties of UiO-66^10,11,23–27^. The thermal stability of the UiO-66 could be maintained up to 500°C after defects in the structure were created^28–30^.

Different procedures have been studied to create defects in the UiO-66 structure, such as using inorganic acid^31^, organic acid^25^, modifications in the synthesis method^26^, and conditions^32–35^. Earlier studies have already shown that changes in the synthesis method and conditions are two basic and effective approaches to improve the catalytic properties^21,32–34^. Among the various methods, using ultrasound energy has created unique advantages compared to conventional sources^36^, such as electrical energy or microwave^3,14,37–39^. The results of these comparisons were shown in the review study by Vaitsis et al.^38^. These include reducing the cost of raw materials, uniform dispersion of particles in the reaction medium, as well as producing smaller and more uniform particles. These properties can also increase the specific surface area of the material^26,40,41^. Hu et al. proved that the synthesis time of the MOFs was reduced using ultrasound energy resource compared with the microwave energy resource^42^. In some studies, an ultrasound source has been used for better mixing^3^. After this step, the resulting mixture is placed in a hot bath or autoclave. Due to the high energy caused by the bursting of bubbles, the reaction can occur in less time. The collapse of bubbles can also turn large particles into small ones, which increases the possibility of defects in the boundary between particles and grains or ligands. Homaee et al. investigated the effect of the synthesis method on the catalytic properties of UiO-66, and the results showed that the ultrasound energy source reduced the particle size^37^. Power changes directly with temperature. As the ultrasound power increases, the maximum ambient temperature also increases. As a result, the particle sizes and the defects' quality can be controlled by raising the power^6,38^. As previously mentioned, the structural defects lead to enhancement of catalysis performance^43^. Nevertheless, the improvement in catalytic properties should be such that there is no change in the thermal and chemical properties of the UiO-66 so that this catalyst can be used in harsh operating conditions.

Based on our knowledge, recently, UiO-66 has been employed as a heterogeneous acid catalyst in various reactions due to its unique properties, high thermal and chemical stabilities^17,28,44–49^. However, its performance has not been evaluated in reactions under harsh conditions (T ≥ 400 °C)^21,50,51^. Note that two points are essential to evaluate the UiO-66 performance in a comparatively harsh reaction condition: First, the UiO-66 stability must be examined to select a reaction within its stability range. Second, active and catalytic sites of UiO-66 must be able to participate in the appropriate reaction. As mentioned before, the UiO-66 is thermally stable up to 550 °C^15,16^. Consequently, the proper reaction temperature should be less than 500 °C to ensure UiO-66 activity, like methanol dehydration reaction (T_reaction_ ≤ 500 °C). DME is the major product of this process. This commodity, like hydrogen, has been introduced as a fuel for the future and is regarded as a clean fuel^52–54^.

Additionally, UiO-66 has acidic properties and, consequently, can theoretically be used as a catalyst in this process. This process can be used to examine the ability of the UiO-66 under challenging conditions and be strengthened in future studies.

Previous research revealed that some organometallic frameworks were used as catalysts in the methanol dehydration reaction^5,10,55,56^. However, the UiO-66 catalyst has only been studied by Connelly et al.^55^ and acetic acid was used as a modulator in the production of UiO-66 in their study. The results revealed that the synthesized catalysts had a relatively low specific surface area, and their activity at 350 °C was weak and unstable. Our current study includes multiple goals, which are listed below:

1. Synthesis and study of the physicochemical properties of UiO-66 without the addition of a modulator for use in the methanol dehydration process.
2. Evaluation and preliminary feasibility of this catalyst's development in processes with high-temperature operational conditions.
3. Improving this compound's performance in the methanol dehydration process over prior research.

Physicochemical properties of the synthesized catalysts were studied by different methods such as XRD (X-ray Diffraction), FTIR (Fourier-Transform Infrared Spectroscopy), SEM (Scanning Electron Microscope), particle size distribution (PSD), Brunauer–Emmett–Teller (BET) nitrogen adsorption/desorption isotherms, thermogravimetric analysis (TGA-DTG) and NH_3_-TPD (NH_3_ Temperature Programmed). Methanol conversion tests were performed in a fixed bed reactor at 350 up to 450 °C and atmospheric pressure. Time on stream was conducted for 12 h at 450 °C over the best catalyst. XRD and TG-DTG Analysis evaluated the effect of the stability test on the catalyst structure. This research could be a step forward for applying UiO-66 and other stable MOFs at high-temperature processes.

## Results and discussions

### Physicochemical characterizations

**Figure 1:**
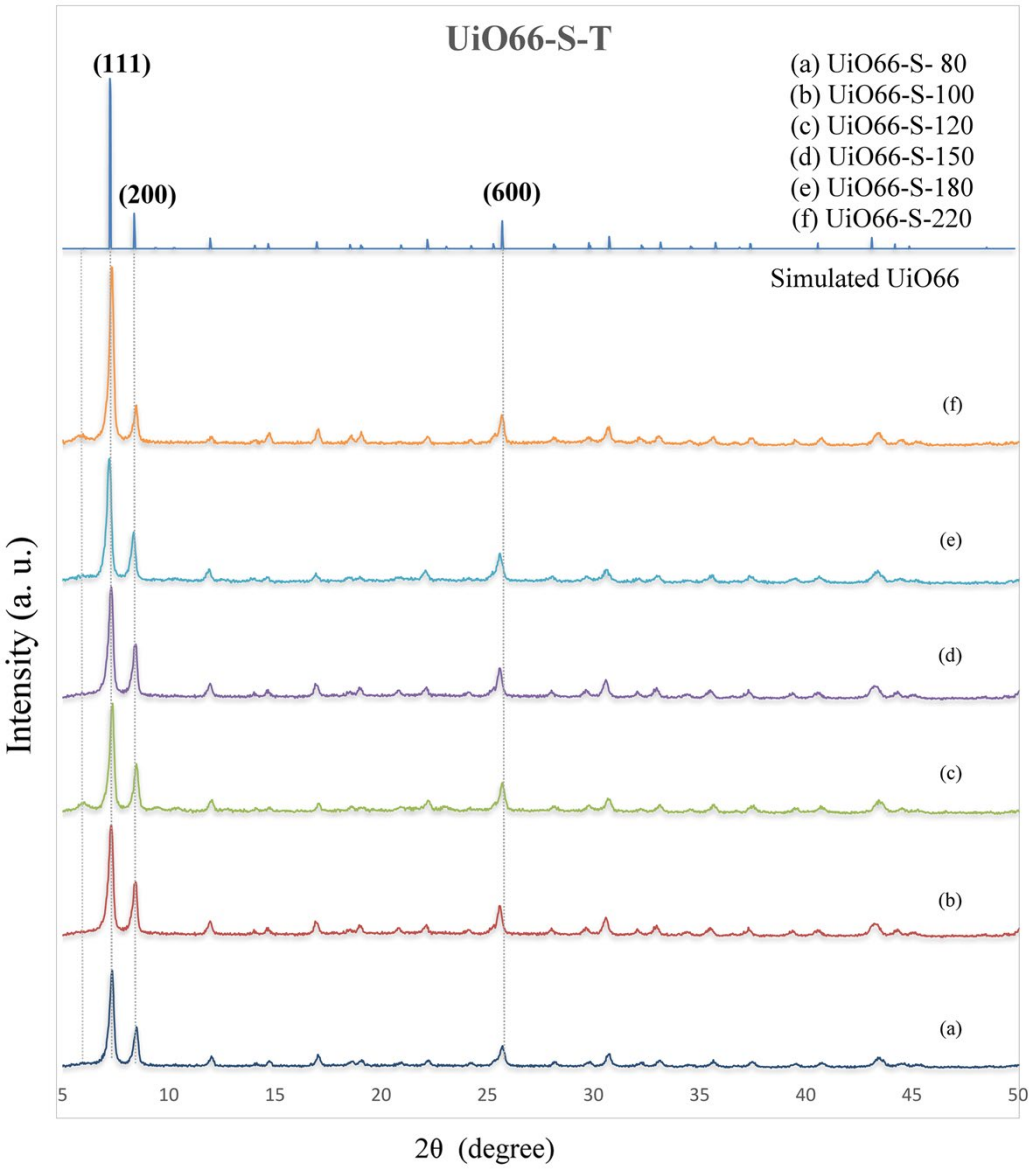
XRD patterns of synthesized catalysts at different temperatures: (**a**) UiO66-80, (**b**) UiO66-100, (**c**) UiO66-120, (**d**) UiO66-150, (**e**) UiO66-180 and (**f**) UiO66-220.

Figure 1 illustrates the XRD patterns of UiO-66 synthesized via the sonochemical-solvothermal method at different temperatures. As can be seen, all samples have a crystalline phase with different intensities and peak areas. The simulated pattern approved the formation of the appropriate phase of UiO-66 (fcu). The results (Fig. 1, Table S1) reveal that increasing the synthesis temperature improves the relative crystallinity and crystallite size of the samples.

Based on the literature, the diffraction peaks at 2θ = 7.4 (111) and 8.5 (002) are the main features of the UiO-66 XRD pattern^16,57,58^. Both are observed in the patterns (Fig. 1a–f). Peak 111 has the highest intensity (2355 a. u.), and an increase in synthesis temperature has the most significant effect, but the other peaks have not experienced significant change.

It is as if a metal cluster (Zr_6_O_6_(BDC)_12_12—SBU) and all bounds have been removed from the corner of the cell unit. It seems that despite the formation of the main phase (with proper integration), the reaction rate between the metal cluster and the ligand has also increased. Each metal cluster should establish 12 bonds with the ligands. Some of these bonds do not form due to the higher reaction rate; thus, it is possible to create the Reo phase via a higher synthesis temperature.

The increasing effect of temperature on the crystallinity of UiO-66 can be seen in Table S1. Crystallinity improves due to the increase in surface area and intensity of prominent diffraction peaks.

Figure S1 shows the FTIR spectra (400–4000 cm^−1^ wave number) of the studied catalysts at different temperatures. These spectra have been recorded in the range of 400–4000 cm^−1^ wave number. The effect of the synthesis temperature on the functional groups can be assessed by comparing the FTIR patterns of the UiO66-T samples, which prove that functional groups are desired in the structure of UiO-66^15,34,45^.

The observed peaks within the spectral range of 500–800 cm^−1^ are attributed to the vibrational modes associated with the bonding of metal oxides. Some bands at 746 and 815 cm^−1^ may be linked to the mixture of C–H vibration, C=C stretching, OH bending, and OCO bending in the terephthalic acid molecule^15^. There are several vibrational peaks before wavenumber 1000 cm^−1^ associated with OH, CH bending, and C=C stretching that overlap with the vibrations of the Zr-O modes^16^. The µ_3_-O and µ_3_-OH stretching vibrations of Zr_6_O_4_(OH)_4_(CO_2_)_12_ can be assigned to the vibrations at 664 and 483 cm^−1^. The asymmetric stretching vibration of Zr-(OC) is described by the absorption band at 551 cm^−1^ in the spectrum of all samples^45^. The asymmetric stretching of CO_2_ surface adsorption is responsible for the weak adsorption at 2360 cm^−1^, though between 1000 and 2000 cm^−1^, some different vibrations can be observed^15^. The BDC (BDC = benzenedicarboxylate) coordination with Zr nodes can be identified as the driver for the band at 1017 and 1506 cm^−1^, while the stretching mode of carboxylate groups is reflected in the two peaks at 1583 and 1397 cm^−1^^16^. The C=O functional groups in the remaining DMF molecules in the UiO-66 structure have an asymmetric stretching frequency of 1658 cm^−1^^3,5,7^. The C–H band in the aromatic ring's plane has an asymmetric stretching frequency of 1155 cm^−1^^15^. All samples show broad peaks in the region of 2800–4000 cm^−1^, which correspond to deformation, symmetric, and asymmetric -OH vibrational modes, which are caused by physisorbed water and –OH groups on the outer surfaces of the microcrystals^16^.

**Figure 2:**
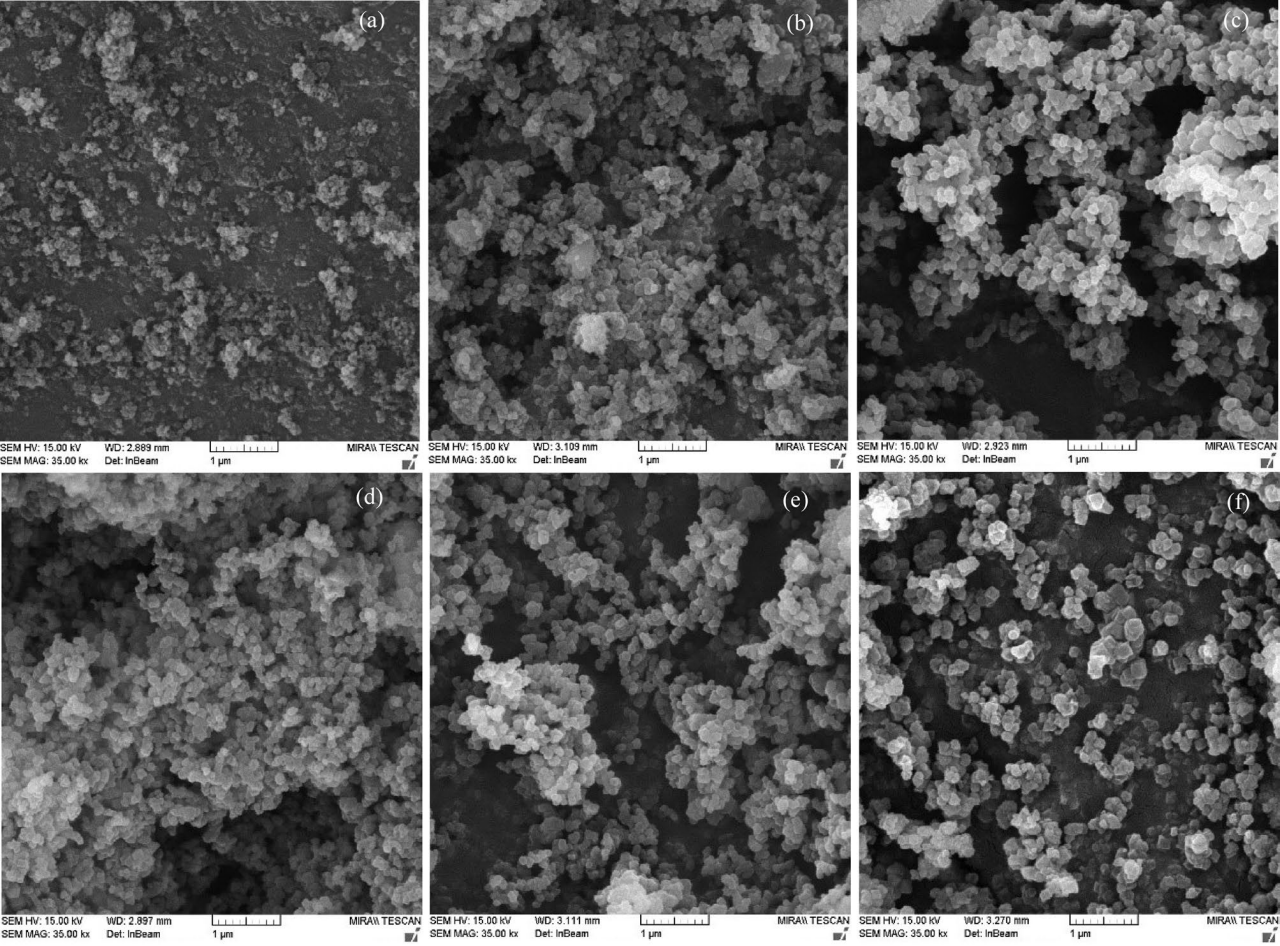
SEM images of synthesized catalysts at different temperatures: (**a**) UiO66-80, (**b**) UiO66-120, (**c**) UiO66-120, (**d**) UiO66-150, (**e**) UiO66-180, (**f**) UiO66-220.

The SEM images (scale 1 µm) of UiO-66 synthesized by the ultrasonic solvothermal method at different temperatures are shown in Fig. 2. It can be seen that the crystallization quality improves with increasing synthesis temperature, as all samples are octahedral crystals in their morphology^17,40^. Thus, the fine structure of the catalyst particles at a temperature of 220 (UiO66-220) is larger than that of the other samples. These observations agree well with the XRD pattern results, the peaks intensity and crystallite size increasing at higher synthesis temperatures slowly. SEM Images also show that particle agglomeration is affected by rising synthesis temperature. At lower synthesis temperatures, the particle size is smaller and the surface energy of the particles is expected to increase, leading to more agglomeration^59^.Some previous studies have shown that particle size uniformity is one of the advantages of using ultrasonic energy in material synthesis^26,60^. Thus, it seems that the application of ultrasonic energy is an important reason for the uniform particle size distribution of all samples. In addition, there is a possibility that some weak bonds, such as the benzene-carboxylate bond, are broken as the intensity and duration of the ultrasonic energy increases. As a result, the ligand is not completely attached to the metal cluster and the particles have not grown.

Image-J analyser was used to analyse the particle size distribution of the UiO66-80 and UiO66-220 catalysts. In addition, higher synthesis temperatures lead to larger particles as shown in Fig. S2a, b. Smaller particles in samples (a) and (b) had particle diameters of less than 200 and 400 nm, respectively. As noted earlier in the X-ray analysis, smaller particles sizes caused to lower intensity peaks in the X-ray analysis. UiO66-80 has a smaller particle size due to the low synthesis temperature, which provides the primary energy for particle formation.

**Figure 3:**
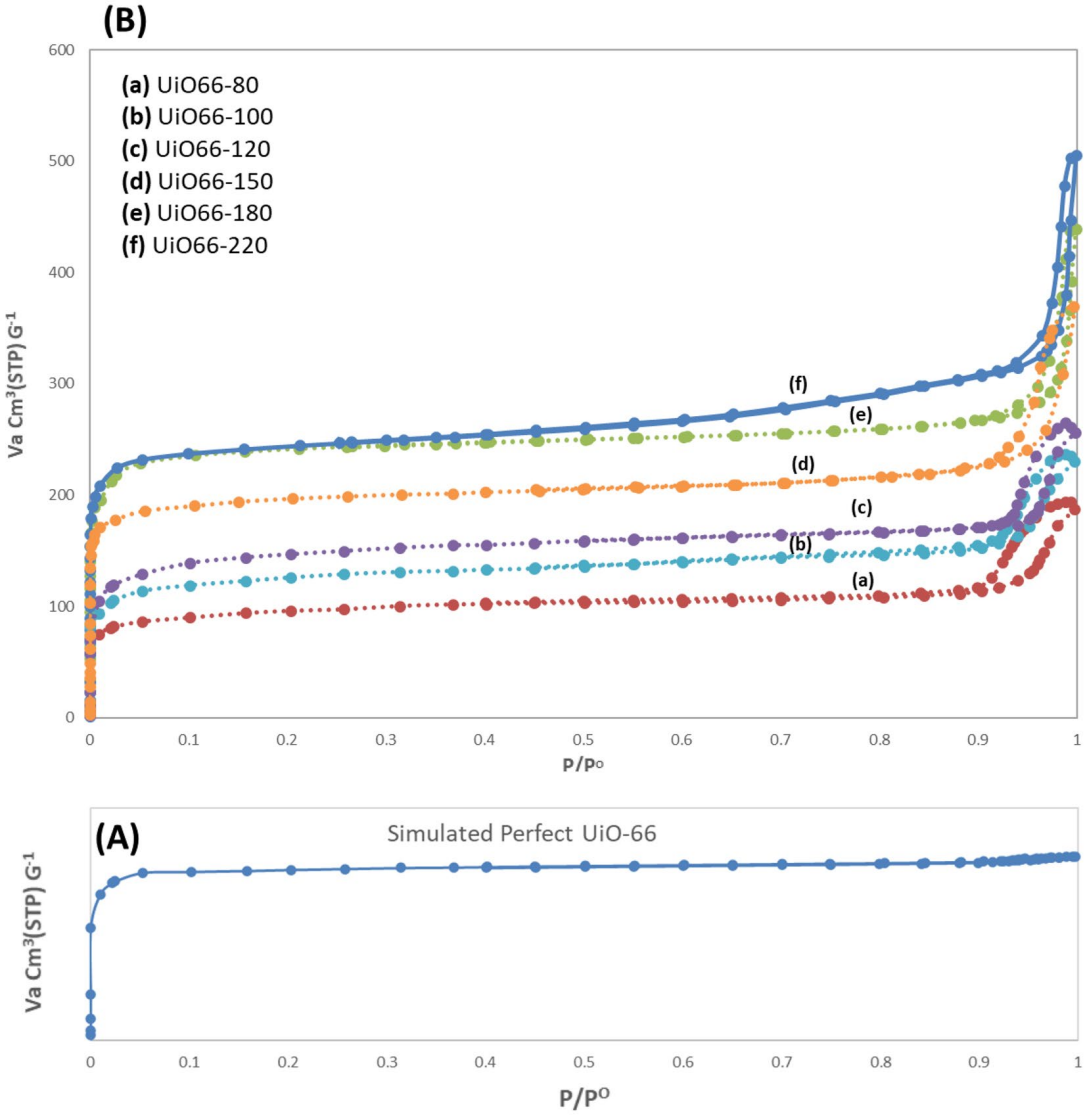
(**A**) Simulated perfect micropore UiO-66 (defect-free), (**B**) N_2_ adsorption–desorption isotherms of synthesized catalysts at different temperatures (80 to 220 °C).

Figure 3A, B shows the N_2_ adsorption–desorption isotherms of simulated perfect micropore UiO-66 (defect-free) and UiO-66 synthesized at different temperatures, respectively. According to the IUPAC classification, the synthesized UiO-66 catalysts were a mixture of types I and IV adsorption–desorption pattern for microporous and mesoporous materials, respectively. Previous studies have shown that UiO-66's ideal micropore structure had the type I adsorption–desorption patterns (Fig. 3A). Also, a combination of type I and type IV patterns occurred due to increasing defects in the structure and the formation of larger cavities in mesoscale^32,33,35^.

Figure 3 shows that when the synthesis temperature increased, the adsorption–desorption pattern transformed to type 4. The results show that all samples had hysteresis loops (between P/P_0_ = 0.4 and 1). The hysteresis loop became thinner and more stretched at higher synthesis temperatures, possibly due to the more uniform size and pore diameter of the channels. The cavities have a well-defined cylindrical pore channels channel according to the standard.

At lower temperatures, the loop tended to have rather irregular pores of hysteresis type H1 because the low temperature did not provide the necessary energy for the reaction between the cluster and the ligand and reduced the structural order. It is also possible that certain ligands prevented the formation of cavities due to defects in the structure, as confirmed by the low specific surface area of the samples at low temperatures. While microcavities are present in the structure and it is also evidenced by the knees and rapid uptake of the N_2_ adsorbed/desorbed pattern at relatively low pressure. As the height of the section increases, the micropore volume also increases. This phenomenon increased the specific surface area of the catalysts, so that their specific surface area has jumped from 330 to 952 m^2^/g. The quantitative results of N_2_ adsorption–desorption isotherms (BET analysis) are presented in Table 1. As can be seen, the rising synthesis temperature has increased the specific surface area and the pore diameter.

**Table 1:** Porosity properties of UiO-66 with different synthesis temperatures.

	S_BET_ (a, BET) (m^2^ g^−1^)	Total pore volume (*p*/*p*_0_ = 0.990) (cm^3^ g^−1^)	V micro pore (cm^3^ g^−1^)	Mean pore diameter (nm)
UiO66-80	330.31	0.29	0.22	2.45
UiO66-100	573.54	0.35	0.23	2.46
UiO66-120	640.06	0.41	0.25	2.47
UiO66-150	827.38	0.57	0.32	2.76
UiO66-180	937.87	0.68	0.36	2.93
UiO66-220	952.12	0.78	0.38	3.36

**Figure 4:**
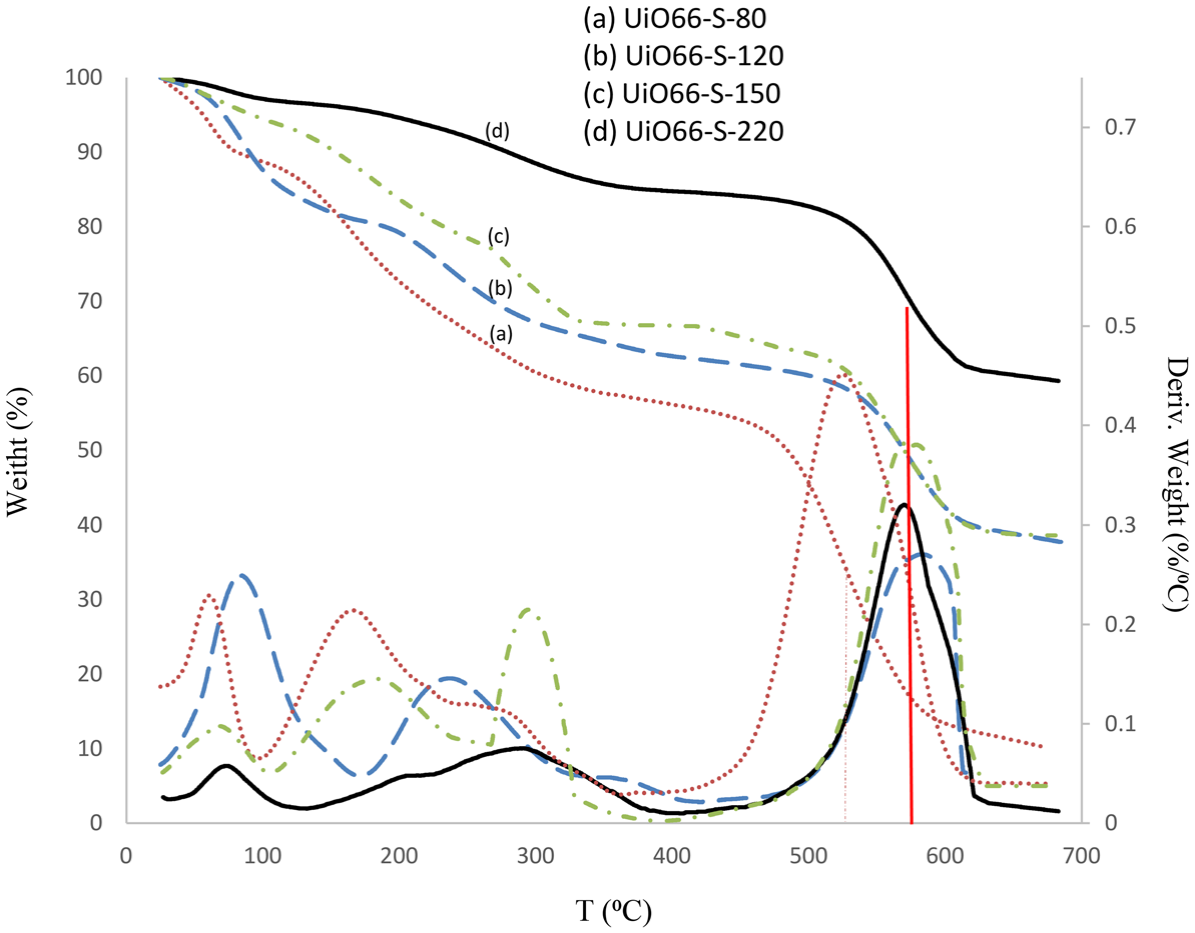
Thermal properties (TG-DTG) of synthesized UiO-66 at different temperatures.

TG-DTG analysis was used to evaluate the thermal stability of catalysts. Figure 4 illustrates the weight loss profiles and weight loss rate. Three-stage weight loss occurred on UiO-66 catalysts^15,34^. The adsorbed water in UiO-66 is lost in the structure at the first stage, before 150 °C, and the second weight loss, at 150–400 °C, is due to DMF degradation and/or ligand evaporation, which was not exchanged with ethanol^34,61^. The Zr_6_O_4_(OH)_4_ nodes are dehydrated to Zr_6_O_6_ at 350 °C during this stage, and water molecules are formed by the removal of OH groups. The decomposition of UiO-66 to ZrO_2_ is responsible for the higher temperature^16^.

All samples remained stable up to 580 °C, except UiO66-80, which was stable up to 520 °C. The TG-DTG analysis revealed that as the synthesis temperature increased, the thermal stability of the samples also increased, so the UiO66-220 had the highest thermal stability. These results showed that weight loss in response to changes in synthesis temperatures has the same trend as thermal stability. As shown in Table S2, the UiO66-220 has the lowest weight loss in all three regions, and the BET analysis showed that the pore diameter and volume increased as defects in the structure increased. So, in the drying and extraction steps, the removal of water and other compounds from the structure becomes more feasible, especially in stages 1 and 2.

Further analysis of weight loss patterns in region 3 reveals that the catalyst structure is made up of more zirconia and ligands. At higher synthesis temperatures, the metal–ligand reaction was better controlled, which resulted in better crystallinity in the samples, as evidenced by XRD analysis. In a complete reaction with the metal cluster, each ligand forms four bonds^29,44,62^. If the number of bonds decreases, the probability increases that other bonds break at higher temperatures; at low synthesis temperatures, the energy required to form four bonds is unavailable, and the number of ligands with no complete bond increases. As a result, the weight loss of samples synthesized at lower temperatures is higher, and the ligand leaves the structure faster. Despite the high content of ligands, these samples have fewer bonds as well as metal clusters in the structure, and they will have a high weight loss after exposure to a process at high temperatures^63^. Hao et al. found that TG analysis would show greater weight loss if ligands did not completely react via metal clusters^34^.

**Figure 5:**
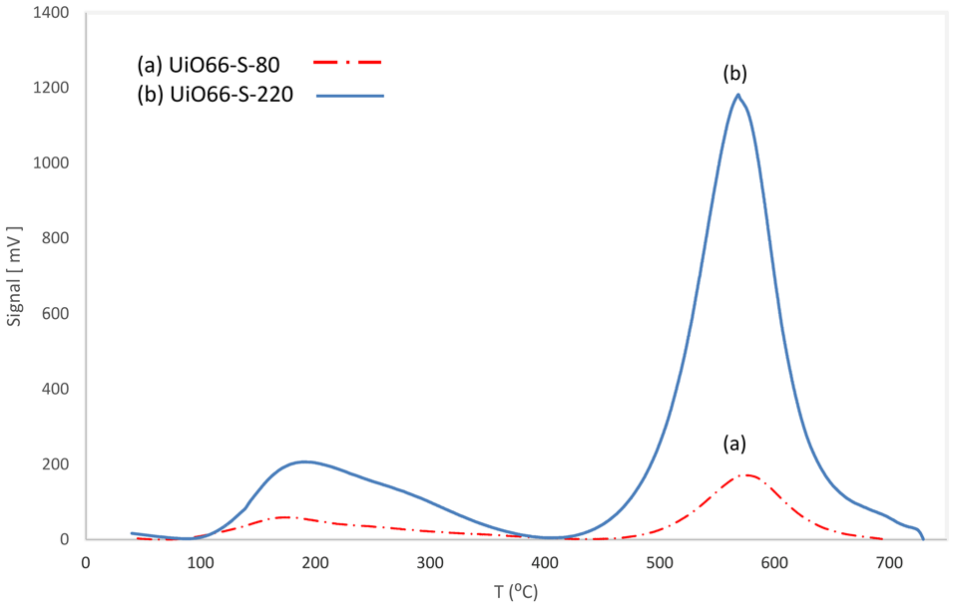
TPD-NH_3_ analysis of UiO-66 synthesized at different temperatures red; 80, and blue; 220 °C.

The acidic properties of the UiO66-80 and UiO66-220 samples were evaluated by NH_3_- TPD analysis. As shown in Fig. 5, two major ammonia desorption peaks are observed in the regions before 400 °C and 400–700 °C; moderate and strong peaks, respectively. Mounfield et al. have demonstrated that a strong peak is associated with defects in the structure^19^.

More detailed results can be found in Table S3. The results show that changing the synthesis temperature from 80 to 220 °C improved the acidic properties. The amount of absorbed ammonia in the UiO66-220 sample was about five times higher than that in the UiO66-80 sample. Based on the results of a previous analysis, the higher specific surface area, larger pore diameter and larger pore volume of UiO66-220 compared to UiO66-80 may be another reason for the increase in ammonia absorption and improvement in acidity (see BET analysis).

## Catalytic performance study toward methanol conversion

**Figure 6:**
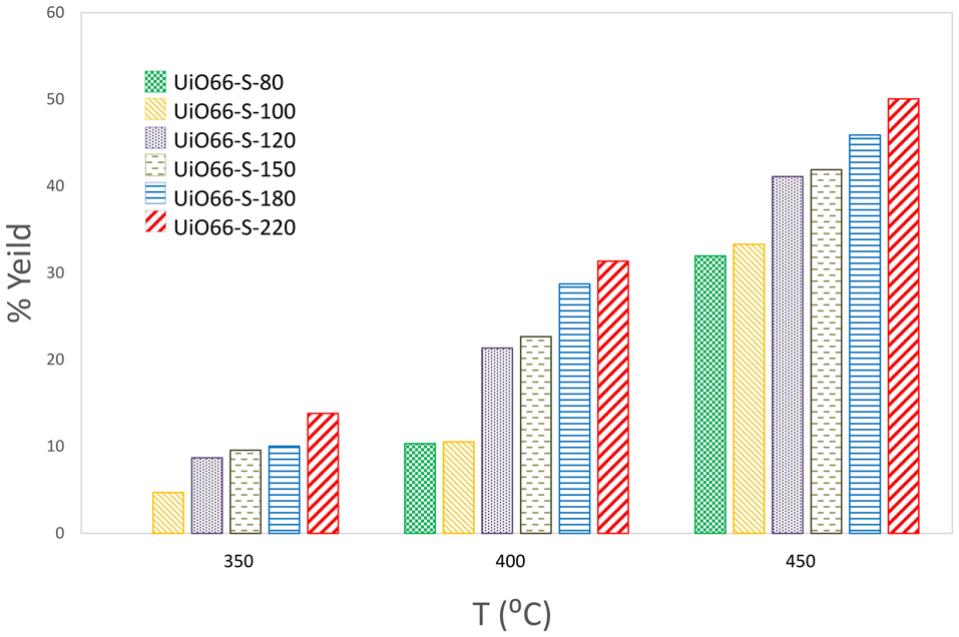
Effect of synthesis temperature on DME yield (process temperatures: 350 up to 450 °C).

The synthesized catalysts were tested for dehydrating methanol in a range of 350–450 °C. Figure 6 and Fig. S3 show the DME yield and methanol conversion of the catalysts, respectively. Examination of the results generally proves two main findings: First, the catalysts synthesized at higher temperatures have higher methanol conversion and better DME yield. Second, the catalyst activity is improved by increasing the reactor temperature. From these results, it is easy to see that the UiO66-220 sample at 450 °C has the best performance with 38% feedstock conversion and 51% DME yield. The activity of various catalysts was investigated in previous studies, and the activity of the catalysts was improved by increasing the reaction temperature from 200 to 450 °C^64–67^. There are two reasons for improving catalyst performance by increasing the synthesis and reactor temperatures:

The first reason is related to a structural property; DME production from methanol is a structure-dependent reaction^64^. The results of the analysis in the previous sections have shown that the increase in the synthesis temperature due to a structural defect causes the formation of the Rho phase to a greater extent. As a result, the acidic property of the catalyst also increased, which agreed well with TPD -NH_3_. Note that acidic sites are the main cause of methanol dehydration. The UiO66-220 catalyst had the highest specific surface area and the largest average pore diameter; therefore, adsorption and diffusion of feedstock and product are enhanced, and higher activity is expected compared to other samples.

Previous studies have shown that methanol conversion involves two basic steps^64,68,69^:

(1) Dehydration of methanol over weak and medium acid sites, and (2) conversion of DME to olefin and higher hydrocarbons responsible for strong acid sites. Despite the strong acid sites, these sites in zirconia metal–organic frameworks tend to absorb more water than methanol^68,70^. Therefore, methanol conversion proceeds only to the dehydration stage.

The second category of reasons is thermodynamic and kinetic problems. According to Arrhenius' law, the reaction rate constantly increases with rising reactor temperature. An equilibrium condition does not exist because the products are continuously removed. Thus, the evolution of the reactor temperature has improved the performance of the catalysts. The results follow previous studies.

**Figure 7:**
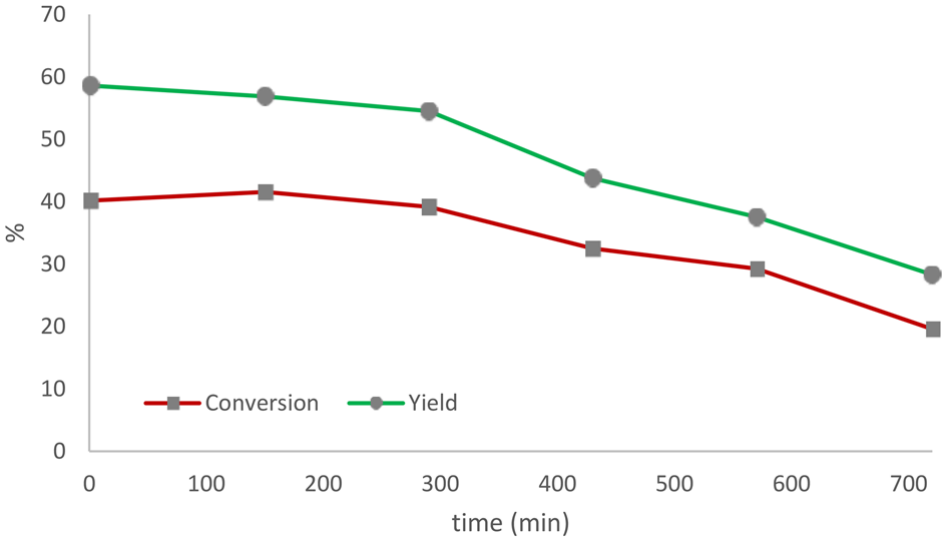
Time on stream performance of UiO66-220 during 12 h; 450 °C.

The catalytic activity of the best catalyst (UiO66-220) was tested for 720 min at a constant temperature (450 °C) and WHSV (weight hourly space velocity). The methanol conversion and DME yield are shown in Fig. 7. First, the fresh catalyst was added to the reactor, then activation was performed, and finally, methanol was fed into the reactor. As it turned out, the activity of the catalyst remained stable for about 300 min. The methanol conversion and the yield of DME remained constant at 40 and 58%, respectively, and gradually decreased after 300 min.

The primary reason for the deactivation of the catalyst is structural degradation over time. The existence of water in the reaction medium may challenge the hydrothermal stability of UiO-66. As mentioned earlier, water molecules tend to adsorb on acidic sites^67,68,70^. Jiao et al. have shown that water, when adsorbed onto the organometallic framework of UiO-66, can cause cleavage of the metal–ligand bond and eventually remove the ligand from the framework^12^.

**Figure 8:**
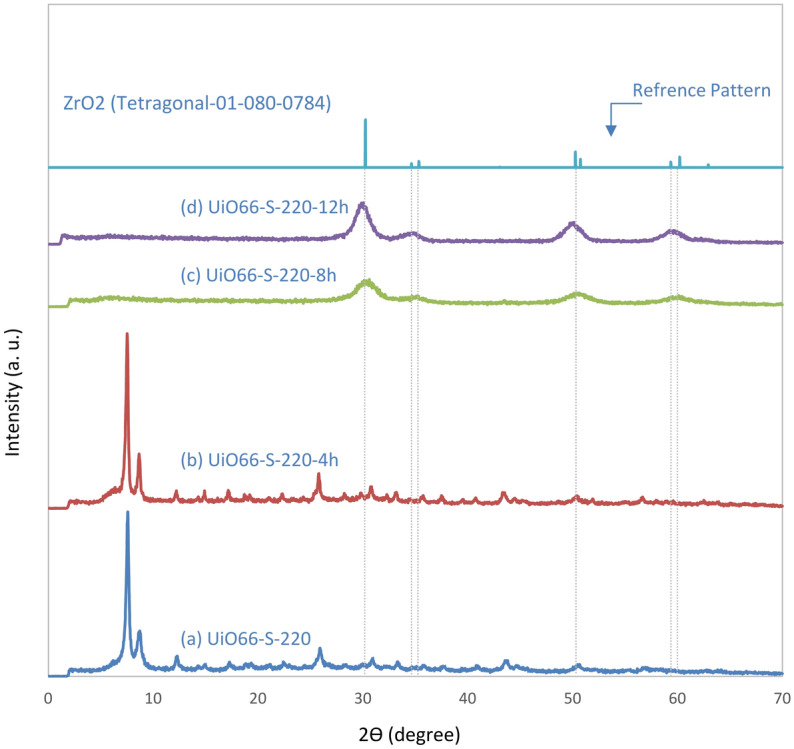
XRD analysis of fresh and spent UiO66-220 catalyst at 4, 8, and 12 h time on stream.

The TG analysis of fresh UiO-66 showed that ZrO_2_ is formed after the removal of the ligand. The formation of zirconia over time in the reaction atmosphere was demonstrated by X-ray analysis (Fig. 8). Coke formation over time is another reason for catalyst deactivation. Some previous studies have demonstrated the coke formation on the surface of acid catalysts in the methanol conversion process^64,67,69,71^. Figure S4 shows the TG analysis in an air atmosphere to evaluate the coke deposition. Moreover, the white of the catalyst was completely black after 12 h of activity. However, the colour of the UiO66-220 catalyst changed slightly to light gray after 5 h, indicating that coke formation was very low until the fifth hour of the reaction.

Several MOFs have been employed as methanol dehydration catalysts in previous studies^5,10,55,56^.

Our analysis revealed that Connelly et al.^55^ carried out a study that was comparable to ours in 2022.

In this work, UiO-66 (UiO-66aa) was synthesized using acetic acid as a modulator. Additionally, the UiO-66aa catalyst's performance in the methanol dehydration reaction was studied in the 200–300 °C range. Table 2 reviews and compares our research's outcomes. Despite the greater reaction temperature, analysis of the data reveals that the catalyst developed in our study (UiO-66) exhibited more activity and performance stability in the reaction. Table 2 shows that the UiO-66 had activity and stability up to 40% as well as a 300 min stability duration at 220 °C. In contrast, the activity of the UiO-66aa catalyst was less than 10% conversion and unstable. The following explanations explain for these differences:

**Table 2:** A comparison of the findings from studies comparable to ours.

Cat	Modulator	Surface area (m^2^ g^−1^)	T_Reactor_ (°C)	P (atm)	Conversion	TOS (min)	The stability time (min)	Ref
UiO-66	–	952	450	1	< 45	720	300	Current study
UiO-66aa	Acetic acid	< 50	300	1	< 10	1000	0	^55^

*aa* acetic acid (modulator).

1. The phase and structure of UiO-66 were altered by the addition of acetic acid. Consequently, different physical, chemical, and thermal characteristics have been observed in comparison to the basic UiO-66.
2. The specific surface area of UiO-66aa has been much less than that of UiO-66, resulting in a lower performance.

Certainly, these results are not desirable, but they can be a beginning point for future investigations and research in order to increase and improve the performance of UiO-66 in the methanol dehydration process.

The XRD patterns of the fresh and spent UiO66-220 catalyst at different times in the stream (fresh (a), 4 (b), 8 (c), and 12 h (d)) are shown in Fig. 8. At the top of the patterns is the reference pattern of tetragonal ZrO_2_ (JCPDS: 01–080-0784) as shown by the diffraction peaks at 2θ = 30.23, 34.6, 35.2, 50.2, and 60.2. Samples (a) and (b) correspond to the UiO-66 pattern, but samples (c) and (d) show the formation of the tetragonal phase ZrO_2_. X-ray diffraction shows that the catalyst maintained its structure until the fourth hour and the crystallinity did not significantly change.

After eight hours of reaction time, the ligand is removed and then the tetragonal phase of ZrO_2_ is formed (as already mentioned in the TG-DTG analysis). The decay of the catalyst structure starts between the fourth and eighth hour. The stability test confirms this phenomenon: the activity of the catalyst decreased after 5 h. Evaluation of the XRD patterns of samples c and d show that the intensity and sharpness of the diffraction peaks increased with rising reaction time, suggesting higher crystallinity. The tetragonal phase of zirconia has more acidic and catalytic properties than the other phases of this material^72^. Thus, it can participate in the methanol dehydration reaction.

Figure S4 shows the TG-DTG results of the spent catalyst after the stability test at 450 °C and 12 h. The TGA-DTG curves show three stages of weight loss: 30–250, 250–400, and 400–650 °C. The first stage can be attributed to the removal of physically adsorbed water^15^. The second stage, with a weak weight loss, may be related to the restructuring of the remaining nodes or the evaporation of the remaining ligand in the structure^16,34^. The X-ray pattern of UiO66-220 proves that if these compounds are present in the structure, their amounts are tiny and/or present in an amorphous phase, because no specific peak was observed. The last step, 400–650 °C, is attributed to the coke residue over the catalyst, which appeared at 502 °C^71,73^. Moreover, the total coke content in the catalyst was about 10%.

## Conclusions

In this study, an attempt was made to find a new approach for the catalytic application of UiO-66 under harsh operating conditions. UiO-66 with high thermal stability (T ≥ 500 °C) and fine acidity was used as catalyst in the methanol conversion process (T ≤ 450 °C).

The catalysts were synthesized by an ultrasound-assisted solvothermal method at different temperatures (80–220 °C). The results showed that the crystallinity, thermal stability, specific surface area, and acidity were improved by increasing the synthesis temperature. The reactor test also showed that the activity of the catalysts increased at higher reaction temperatures. Among the results, UiO66-220 exhibited the highest conversion (38%) and product yield (51%—DME) at 450 °C. Due to the strength of UiO-66 acid, the reaction continued to the dehydration stage. The study of the stability test for 12 h at 450 °C showed that the UiO-66 was stable for about 5 h at 220 °C.

## Materials and methods

Metal precursors, Zirconium tetrachloride (ZrCl_4_, 98%) and terephthalic acid ( H_2_BDC, 99%) were supplied by Sigma-Aldrich. Also, N, N-dimethylformamide (DMF, 99%), methanol, and ethanol were purchased from Loba Chemie (laboratory reagent & fine chemicals-India). All chemicals were used without further purification.

Figure S5 (Supplementary Information) shows the preparation steps of UiO-66 at different temperatures via the Sonochemical-Solvothermal method. In brief, ZrCl_4_ (0.7 g, 3 mmol) and terephthalic acid (BDC, 0.5 g, 3 mmol) were dissolved in 50-ml DMF at room temperature for 20 min, respectively. The obtained solution was mixed and sonicated for 60 min in 100 W. The mixture was then poured into a 150 ml Teflon-liner stainless steel autoclave and heated at different temperatures (80 to 220 °C) for 24 h. The obtained white product was centrifuged, washed with DMF (3× for 24 h), and suspended in ethanol for one day. After DMF to ethanol exchange, the powder was centrifuged again and dried at 200 °C for 6 h. Table S4 lists the nomenclatures of samples (UiO66-T; T = 80–220 °C).

Different techniques were used to evaluate the physicochemical properties of the catalysts. These characterizations are XRD, FTIR, N_2_ Adsorption–desorption isotherm (BET), TGA, SEM, and TPD-NH_3_. X-ray diffraction (XRD) assessments were carried out using an Intel EQUINOX 3000, USA with Cu Kα radiation in the 2θ range of 5°–50° (λ = 1.5406 Å). Infrared (IR) spectra were recorded on an AVATAR-Thermo (USA) Fourier transform infrared (FTIR, 400–4000 cm^−1^). Thermogravimetric analysis data were collected on TGA-DTG SDT Q600 V20.9 Build 20 with a heating rate 10 °C/min from 25 to 700 °C under argon atmosphere to evaluate the thermal stability. Nitrogen sorption isotherms were measured using a BELSORP MINI II- BEL Japan at − 196 °C (evacuation at 300 °C for three h and P/P_0_ = 0.05–0.35) for the determination of specific surface area and pore volume. Also, SEM images were obtained using MIRA III TE-SCAN to observe the morphology of the samples. Temperature-programmed desorption of ammonia (NH_3_-TPD) was used to investigate the acid properties of the catalysts.

Figure S6 shows the schematic of the experimental setup for the methanol to DME process. This setup had three sections, an activating section, a chemical reaction section, and analytical equipment to measure the catalyst activity. First, catalyst activation was carried out in an N_2_ atmosphere at 350 °C for one hour. Next, a syringe-pump fed pure methanol to a tubular fixed-bed reactor (stainless-steel; ID: 8 mm). The reactor was loaded with 0.25 g of the catalyst for each test. The catalyst activity was investigated as a function of temperature (350–450 °C) at WHSV = 6 h^−1^ and atmospheric pressure. Also, Time-on-stream (TOS) behaviour over the most active catalyst was evaluated during 12 h at 220 °C. Gas chromatography (Agilent, USA, 6890 N) analyzed the product. The was equipped with a Plot-Q column (Agilent) and a TCD and FID detector. A sample of GC analysis was presented in Fig. S7.

### Supplementary Information

Supplementary Information.

## Data Availability

The datasets used and/or analyzed during the current study are available from the corresponding author on reasonable request.
