# Supplementary material for: Production of eco friendly DME fuel over sonochemically synthesized UiO66 catalyst
Source: Sci Rep. 2024 Jan 19;14:1755. doi: 10.1038/s41598-024-52155-8 (PMC10799073; doi:10.1038/s41598-024-52155-8)
Supplement: Supplementary file 1 — Supplementary Information. [file 41598_2024_52155_MOESM1_ESM.pdf]

Supplementary Information

Optimum catalyst selection in clean fuel production: A case study of UiO66 performance check by operational and synthesis temperature variations

Mahdi Sharifi[1], Roein Halladj[1, *], Sima Askari[2]

*1-Faculty of Chemical Engineering, Amirkabir University of Technology (Tehran Polytechnic), P.O. Box 15875-4413, Tehran, Iran. (*Corresponding author: Amirkabir University of Technology, Department of chemical Engineering, No. 350, Hafez Ave, Valiasr Square, Tehran, Iran 1591634311, Tel: +982164543200 , Fax: +982166499066 Email: halladj@aut.ac.ir, (Roein Halladj), https://orcid.org/0000-0001-9887-5435).*

2- Department of Chemical Engineering, Science and Research Branch, Islamic Azad University, P.O. Box 14778-93855, Tehran, Iran

Quantitative data in XRD analysis:

Table S1. XRD quantitative data of synthesized catalysts at different temperatures.

	Peak (111)	Relative Crystallinity*	Relative Crystallinity	Crystallite Size**
**XRD**	~2θ	%	%	
**(a)UiO66-80**	7.4	58.25	34.58	332
**(b)UiO66-100**	7.4	74.71	27.81	330.33
**(c)UiO66-120**	7.4	63.02	78.82	316.66
**(d)UiO66-150**	7.4	71.78	47.53	330
**(e)UiO66-180**	7.4	79.03	76.47	314
**(f)UiO66-220**	7.4	100	100	345.33

* (A_i/A_{220}) *100, **Scherrer Equation

FTIR spectrum:

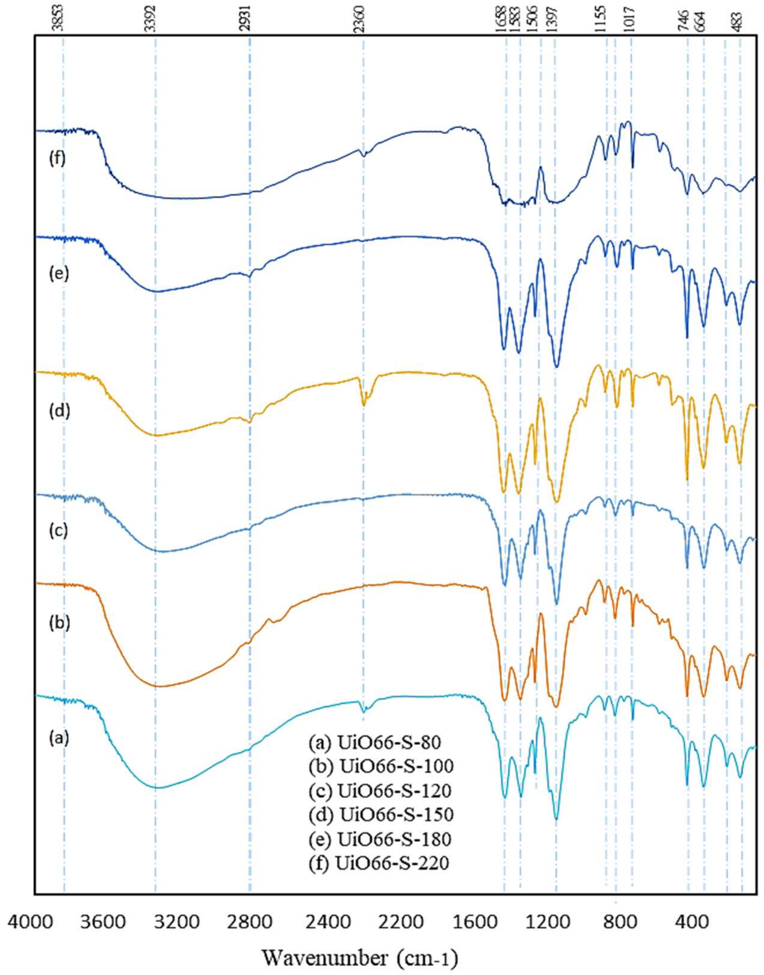

Fig. S1 FTIR spectrum of synthesized catalysts at different temperatures: of synthesized catalysts at different temperatures: (a) UiO66-80, (b) UiO66-100, (c) UiO66-120, (d) UiO66-150, (d) UiO66-180 and (e) UiO66-220.

Particle Size Analysis:

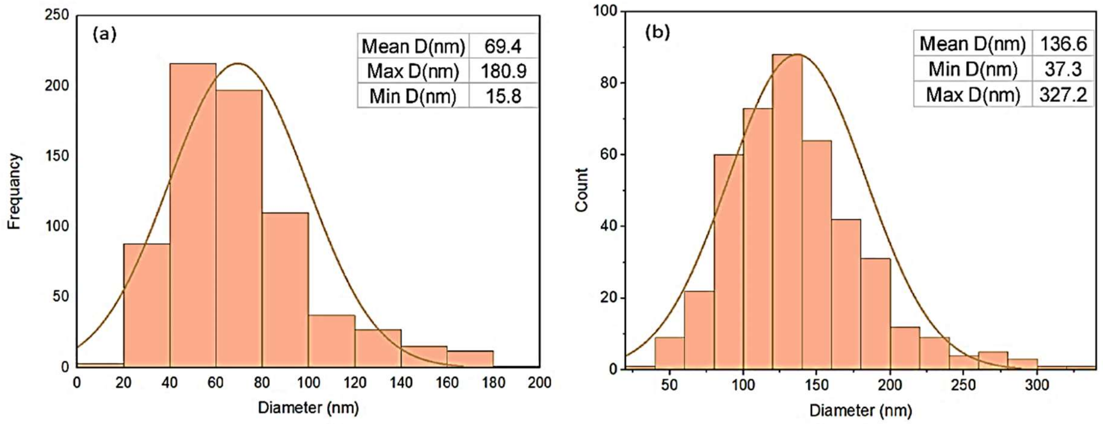

Fig. S2 Particle size distribution of (a) UiO66-80 and (b) UiO66-220

Quantitative data in TGA analysis:

Table S2. weight loss properties of UiO66 synthesized at different temperatures.

T (^oC)	A	B	Weight loss from 25 to 480	Weight loss from 480 to 630	Relative Weight Loss	Relative Weight $Zr_6O_6(C_8H_4O_4)_6$ / 6 ZrO2	Total Weight Loss
**Syn. Temperature**	**% Weight; Read at T(^oC)**	**% Weight; Read at (^oC) T**	**100-A**	**A-B**	**(100-A)/(A-B)**	**A/B**	**100-B**
**80**	57.4; 360	12.13; 630	42.64	45.23	0.94	4.73	87.87
**120**	63.4; 400	39.12; 630	36.58	24.28	1.51	1.62	60.88
**150**	67.04; 350	40.11; 630	32.96	26.93	1.22	1.67	59.89
**220**	85; 380	60.5; 630	15.04	24.51	0.61	1.41	39.52

Quantitative data in NH_3- TPD analysis:

Table S3 Acidic properties of samples based on TPD-NH_3 patterns**;** UiO66-80, and 220.

Sample	Peak area of acid sites		Distribution of acid sites ($mmol_{NH3}$/g)		Total amount ($mmol_{NH3}$/g)
	**Moderate sites T ≤450 °C**	**Strong sites 400-700 °C**	**Moderate sites T ≤400 °C**	**Strong sites 400-700 °C**	
**UiO66-80**	9111.87	71281.26	1.04	1.11	2.15
**UiO66-220**	31685.39	110082.61	1.21	4.08	5.29

Methanol Conversion:

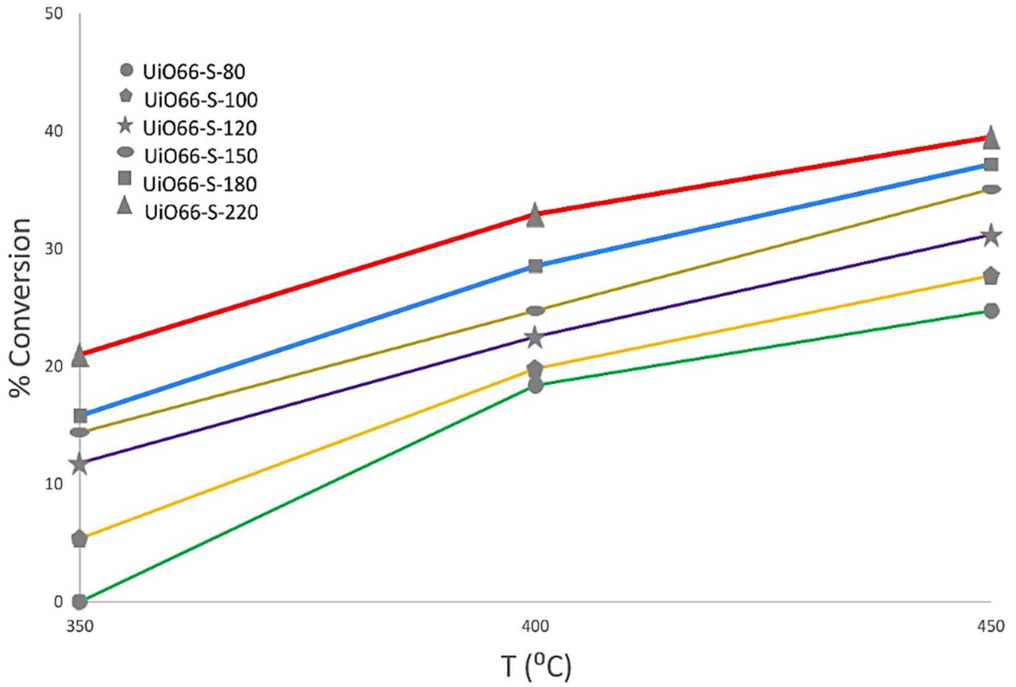

Fig. S3 Effect of reaction temperature on feed conversion (T: 350-450 ºC).

TGA Analysis for best catalyst after stability test

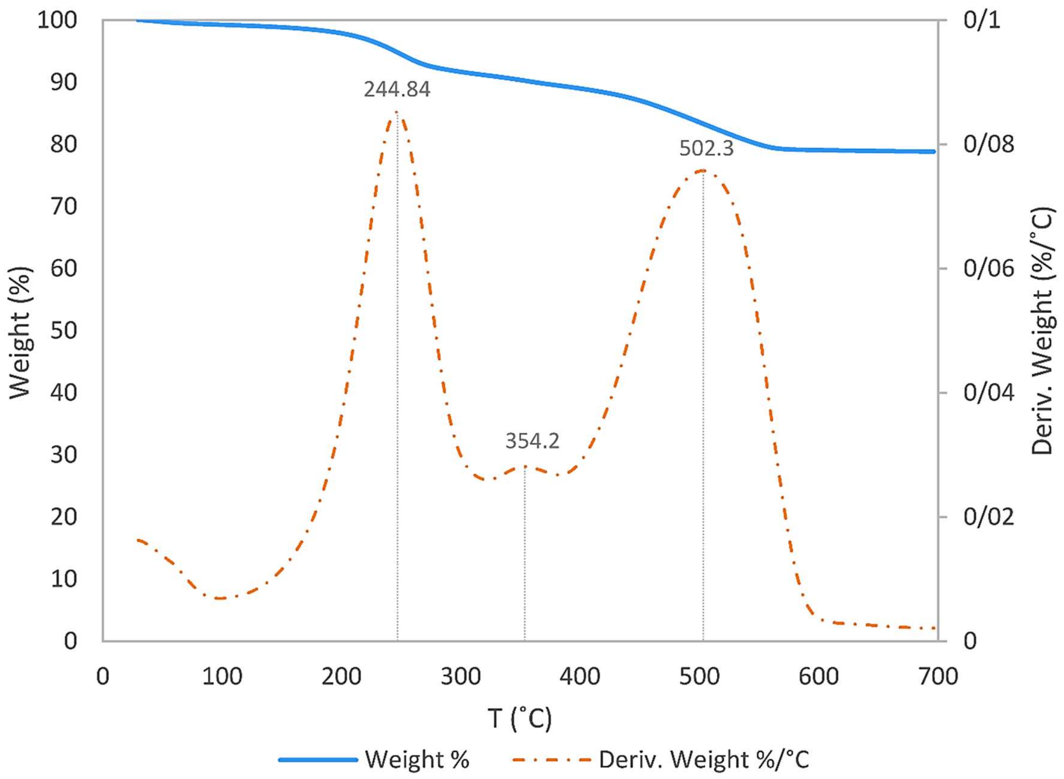

Fig. S4 TG-DTG analysis of UiO66-220 spent catalyst (450 ̊C; 12 h) at Air atmosphere

Synthesis Method

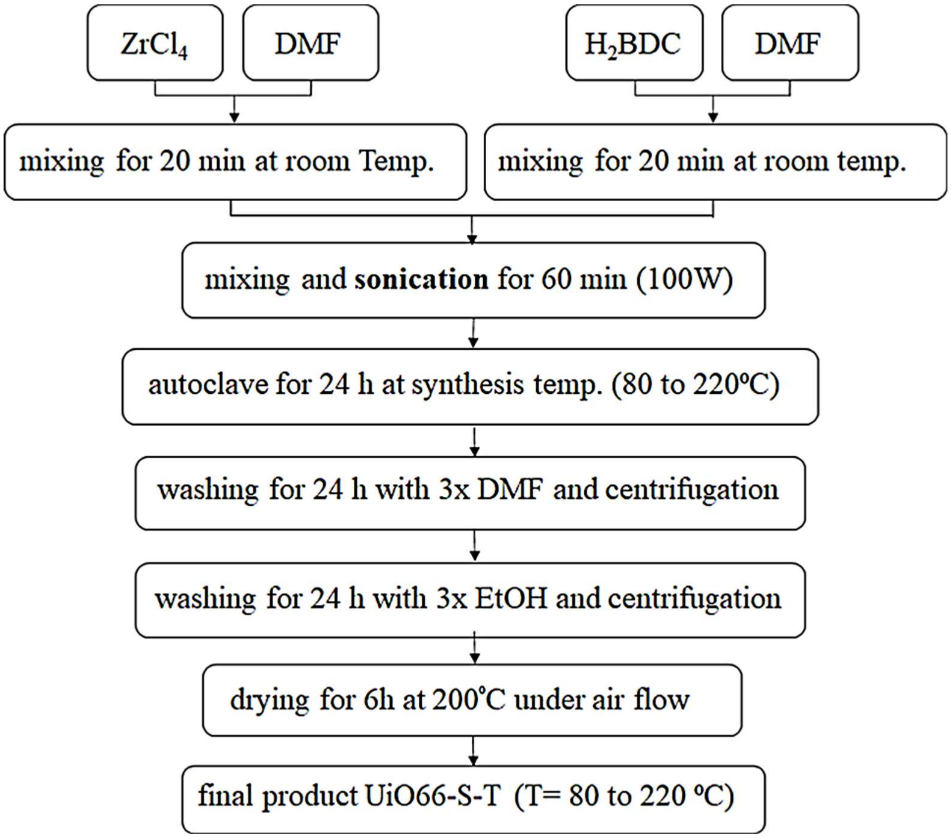

Fig. S5 Different steps of samples synthesis via sonication solvothermal method

Table S4 UiO66 synthesis conditions for different samples

Catalyst (UiO66-T)	Synthesis method	Zr/BDC	time of ultrasound (min)	time (hr)	Synthesis Temperature (°C)
**UiO66- 80**	Sonication-Solvothermal	1	30	24	80
**UiO66-100**	Sonication-Solvothermal	1	30	24	100
**UiO66-120**	Sonication-Solvothermal	1	30	24	120
**UiO66-150**	Sonication-Solvothermal	1	30	24	150
**UiO66-180**	Sonication-Solvothermal	1	30	24	180
**UiO66-220**	Sonication-Solvothermal	1	30	24	220

Eequations:

The conversion and yield for all samples were calculated according to the following equations.

$$\text{Conversion}_{\text{MeOH}}\% = \frac{\text{The mass of methanol consumed}}{\text{The mass of methanol entered}} \times 100$$

$$\text{Yield}_{\text{DME}}\% = \frac{\text{The mass of DME}_{\text{out}}}{(\text{The mass of methanol}_{in} - \text{The mass of methanol}_{out})} \times 100$$

Schematic diagram of experimental setup

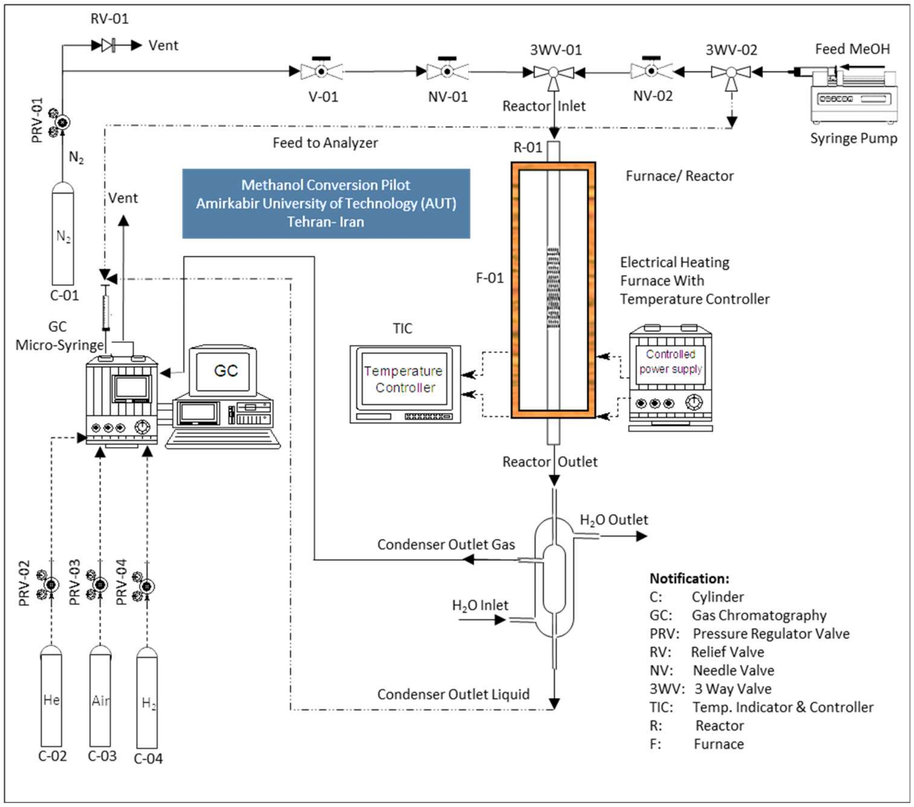

Fig. S6 Experimental setup for evaluation of catalysts toward methanol conversion

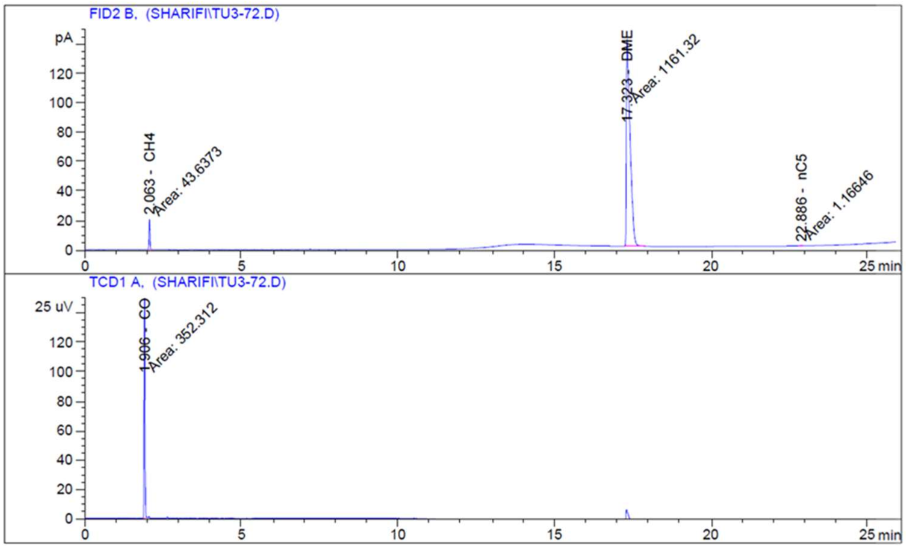

Fig. S7 GC analysis of the gases of UiO66-220 catalyst at 450 ℃.
